# Ectopic BASL Reveals Tissue Cell Polarity throughout Leaf Development in *Arabidopsis thaliana*

**DOI:** 10.1016/j.cub.2018.06.019

**Published:** 2018-08-20

**Authors:** Catherine Mansfield, Jacob L. Newman, Tjelvar S.G. Olsson, Matthew Hartley, Jordi Chan, Enrico Coen

**Affiliations:** 1 John Innes Centre, Colney Lane, Norwich NR4 7UH, UK

**Keywords:** BASL, *Arabidposis*, polarity, tissue cell polarity, planar cell polarity, leaf development

## Abstract

Tissue-wide polarity fields, in which cell polarity is coordinated across the tissue, have been described for planar organs such as the *Drosophila* wing and are considered important for coordinating growth and differentiation [1]. In planar plant organs, such as leaves, polarity fields have been identified for subgroups of cells, such as stomatal lineages [2], trichomes [3, 4], serrations [5], or early developmental stages [6]. Here, we show that ectopic induction of the stomatal protein BASL (BREAKING OF ASYMMETRY IN THE STOMATAL LINEAGE) reveals a tissue-wide epidermal polarity field in leaves throughout development. Ectopic GFP-BASL is typically localized toward the proximal end of cells and to one lobe of mature pavement cells, revealing a polarity field that aligns with the proximodistal axis of the leaf (base to tip). The polarity field is largely parallel to the midline of the leaf but diverges in more lateral positions, particularly at later stages in development, suggesting it may be deformed during growth. The polarity field is observed in the *speechless* mutant, showing that it is independent of stomatal lineages, and is observed in isotropic cells, showing that cell shape anisotropy is not required for orienting polarity. Ectopic BASL forms convergence and divergence points at serrations, mirroring epidermal PIN polarity patterns, suggesting a common underlying polarity mechanism. Thus, we show that similar to the situation in animals, planar plant organs have a tissue-wide cell polarity field, and this may provide a general cellular mechanism for guiding growth and differentiation.

## Highlights

• Ectopic expression of BASL in *Arabidopsis* leaves reveals coordinated polarity
• The ectopic BASL polarity field is independent of the stomatal lineage
• The polarity field reorients around serrations, mirroring PIN1 polarity

## Graphical Abstract

**Figure:**
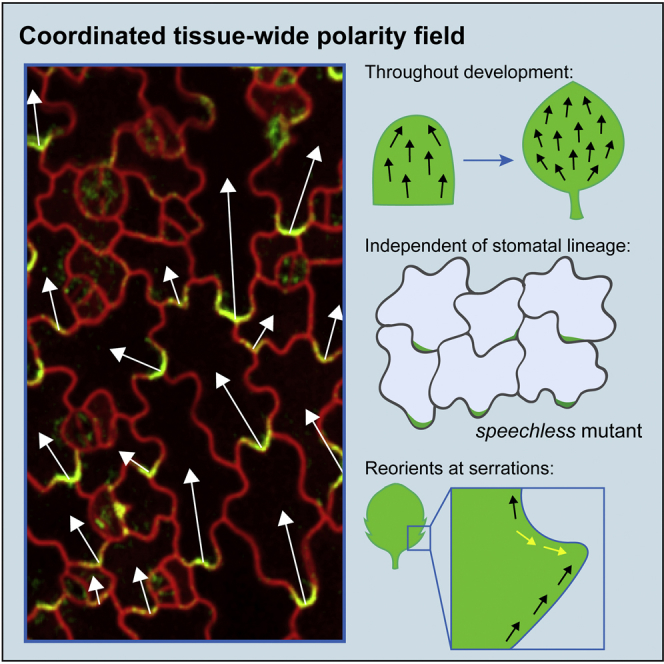

## Teaser

Mansfield et al. demonstrate a coordinated tissue cell polarity field in the *Arabidopsis* leaf epidermis revealed by ectopic expression of BASL. This polarity field is independent of the stomatal lineage and reorients around serrations, mirroring the polarity of PIN1.

## Results and Discussion

### Ectopic BASL Reveals a Polarity Field Independent of Stomatal Lineages

Asymmetries across individual cells (cell polarity) can be coordinated across a tissue to give tissue-wide polarity fields [7]. Polarity fields have been invoked to account for patterns of oriented growth of planar organs, such as leaves [8]. Mathematically, a polarity field corresponds to each position in space having a vector (a vector field) [9]. In biological terms, these positions may correspond to individual cells. However, evidence for a tissue-wide polarity field maintained during planar plant organ development has been lacking.

Several proteins preferentially localized to one end of the cell (i.e., exhibiting cell polarity) have been described in plants, including PIN-FORMED (PIN) proteins, BASL (BREAKING OF ASYMMETRY IN THE STOMATAL LINEAGE), BRXL2 (BREVIS RADIX-LIKE 2), POLAR (POLAR LOCALIZATION DURING ASMMETRIC DIVISION AND REDISTRIBUTION), OCTOPUS, BORs (BORON TRANSPORTERS 1), and NIPs (NODULIN26-LIKE INTRINSIC PROTEINS) [2, 10, 11, 12, 13, 14]. Some of these proteins, notably PIN1 and BRXL2, exhibit polarity coordination in the developing leaf epidermis. PIN1 is preferentially localized at the distal end of cells in leaf primordia, but this pattern disappears at later developmental stages [6, 15]. BRXL2 shows preferential localization to the proximal end of cells in the stomatal lineage [2], compounded by a spiral pattern of polarity switching involved in stomatal spacing [16].

Here, we use BASL to explore polarity patterns in developing leaves. BASL has a well-characterized polarity pattern that is similar to BRXL2, localizing to a crescent in stomatal lineage cells [2] [16]. Localized BASL domains have also been described in root cells ectopically expressing BASL [11].

To see if a polarity field could exist across the leaf independently of the stomatal pathway, we exploited the *speechless* (*spch*) mutant, which lacks stomatal lineages. We induced expression of *35S::GFP-BASL* using a heat-shock-inducible Cre-lox system [17] to avoid potentially pleiotropic effects of overexpressing BASL throughout development [11].

**Figure 1:**
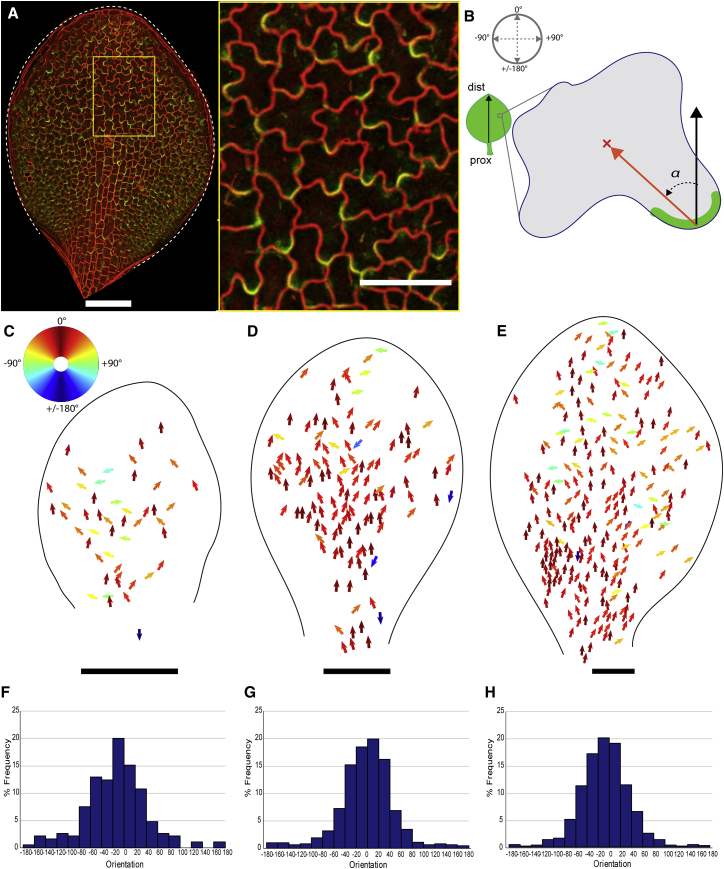
Ectopic BASL Reveals a Polarity Field Independent of Stomatal Lineages (A) Induced *35S::GFP-BASL* in *speechless* leaf stained with propidium iodide (PI). Scale bar is 50 μm in right panel. (B) BASL vectors (orange arrow) assigned from BASL crescent to cell centroid. α between midline vector (black arrow) and BASL vector. (C–E) Ectopic BASL vectors colored according to color wheel (in C) indicating α orientation in *speechless* leaves of (C) 50–200 μm, (D) 200–400 μm, and (E) 400–800 μm width categories. Leaf outlines shown. (F–H) Vector orientation in *speechless* leaves pooled from widths (F) 50–200 μm (n = 185 cells, 4 leaves, σ = 55.34), (G) 200–400 μm (n = 1199 cells, 12 leaves, σ = 49.43), and (H) 400–800 μm (n = 2063 cells, 9 leaves, σ = 44.68). 0° represents proximodistal vector. Scale bars are 100 μm except for the right panel of (A). See also Figure S1.

Ectopically induced BASL was asymmetrically localized in leaf epidermal cells of *spch* (Figure 1A). Signal typically spanned cell vertices (three-way junctions, Figure S1F), allowing assignment to individual cells. In pavement cells, signal was typically observed in a single lobe, toward the proximal end of the cell. To quantify the polarity pattern, we assigned cell unit vectors that pointed from the midpoint of the BASL crescent signal to the cell centroid (Figure 1B). To avoid subjective bias, we randomly rotated automatically segmented single cells before BASL signal was manually identified (Figures S1G–S1O). Processed cells were then returned to their original position and orientation. The BASL vector orientation was calculated with respect to the proximodistal midline vector of the leaf (Figure 1B) and plotted according to a color map (Figure 1C). We refer to the resulting vector field as the ectopic BASL polarity field.

At all developmental stages analyzed, BASL vectors were largely proximodistally oriented in *spch* (i.e., BASL localized toward the proximal end of cells; red/orange arrows in Figures 1C–1E). Some vectors deviated from this proximodistal pattern, particularly toward the leaf tip, though very few vectors pointed proximally (Figures 1C–1E). BASL vector orientations from multiple *spch* leaves were pooled according to leaf size and plotted in histograms (Figures 1F–1H). More than 90% of the BASL vectors were within the range of −80° to 80°. Thus, ectopic BASL reveals a strongly coordinated proximodistal polarity field across leaves of different sizes that is independent of stomatal lineages.

### The Polarity Field Revealed by Ectopic BASL Is Present in Wild-Type Leaves

**Figure 2:**
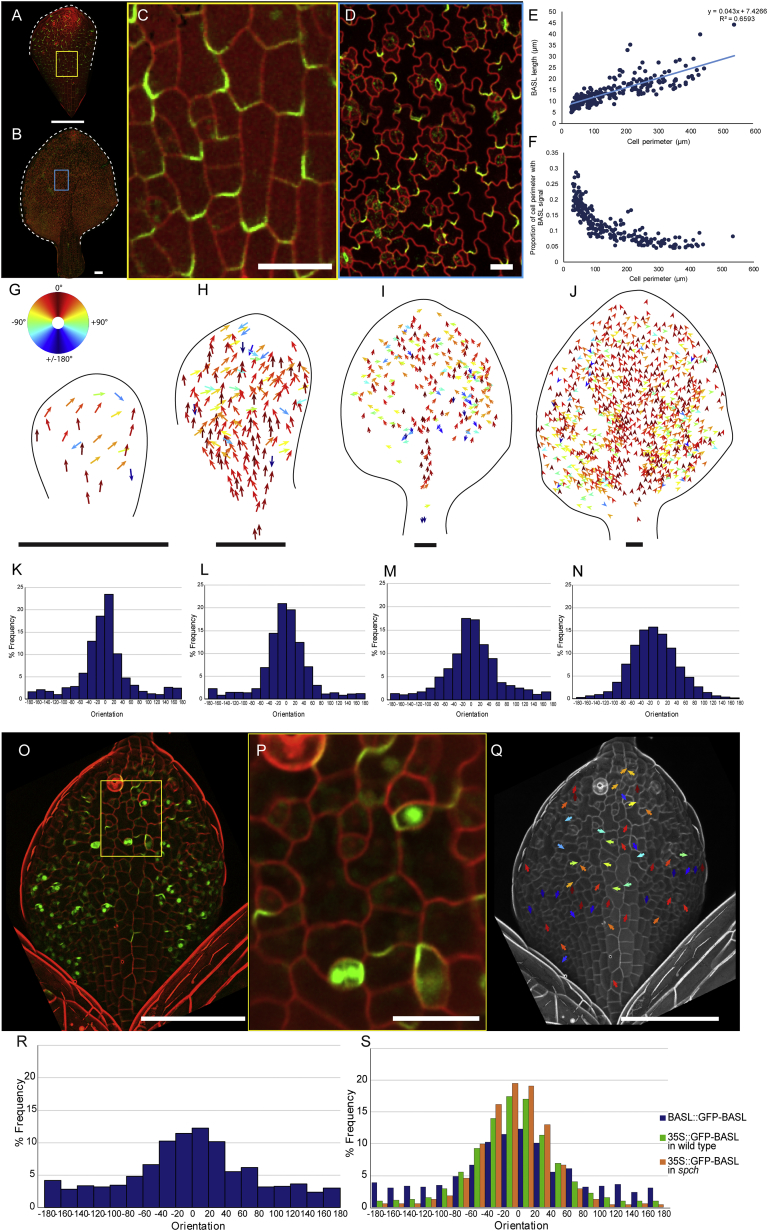
Ectopic BASL in a Wild-Type Background and BASL::GFP-BASL Show Coordinated Patterns throughout Development (A–D) Induced *35S::GFP-BASL* in (A) 50–200 μm and (B) >800 μm width leaves magnified in (C) and (D), respectively. Cell outlines shown using PI staining in C and RFP-PM in D. (E) Length of BASL crescent against cell perimeter for leaves of various sizes. (F) BASL crescent length as a proportion of cell perimeter. (G–J) Ectopic BASL vectors colored according to color wheel (in G) in leaves of (G) 50–200 μm, (H) 200–400 μm, (I) 400–800 μm and (J) 800+ μm widths. (K–N) Vector orientations pooled in leaves of (K) 50–200 μm (n = 1042 cells, 15 leaves, σ = 64.76), (L) 200–400 μm (n = 1464 cells, 9 leaves, σ = 57.25), (M) 400–800 μm (n = 890 cells, 4 leaves, σ = 63.67), and (N) 800+ μm (n = 3642 cells, 4 leaves, σ = 52.71) widths. (O and P) (O) *BASL::GFP-BASL* leaf stained with PI and magnified in (P). (Q) BASL vectors from leaf in (O) colored according to color wheel (shown in G). (R) BASL vector orientations in *BASL::GFP-BASL* pooled from multiple leaves from 50–800 μm width (n = 1319 cells, 21 leaves, σ = 82.3). (S) Percentage frequency of BASL vector orientations for induced *35S::GFP-BASL* in WT and *speechless* background and *BASL::GFP-BASL*. Each genotype pooled from at least 20 leaves from 50–800 μm. p < 10^−5^ for each pairwise chi-squared comparison (Table S1). Scale bars are 100 μm in (A), (B), (G)–(I), (O), and (Q) and 20 μm in (C), (D), and (P). See also Figures S1 and S2.

Given that ectopic BASL reveals a proximodistal polarity field in *spch*, we might expect a similar field to be present in the non-stomatal lineage cells of wild-type leaves. To test this hypothesis, *35S::GFP-BASL* was induced in a wild-type background at different developmental stages (Figures 2A–2D). As in *spch*, ectopic BASL was predominantly observed at the proximal end of cells, often at cell corners (Figure 2C) or within single-pavement cell lobes (Figure 2D). These cells included those above the midvein, which do not develop stomatal lineages [18]. Proximal localization was confirmed from analysis of sectors of BASL expression (Figures S1A–S1E), and by polarity quantification (Figures 2G–2N).

BASL is not normally expressed outside stomatal lineage cells, suggesting that ectopic BASL expression either induces polarity or reveals a polarity field that does not itself depend on BASL function. If ectopic BASL induces polarity, we might expect signal to gradually coalesce on a proximal domain following induction. Time-lapse imaging leaves after heat-shock induction showed that, rather than coalescing, ectopic BASL appeared in its proximal location from approximately 12 hr after heat-shock induction and gradually intensified (Figure S2). This suggests that ectopic BASL does not itself induce cell polarity but rather marks a pre-existing polarity.

We hypothesize that ectopic BASL binds to interacting partners—for example, proteins or lipid domains—that are located proximally in each cell throughout development. We refer to these hypothetical interacting partners as providing a proximal molecular address. Localization of BASL to cell corners or to a single lobe of pavement cells may reflect a single address located at the proximal extrema of the cell. It is also possible that positioning of the proximal address is modulated by factors establishing lobe and neck formation [19, 20, 21] or located at cell corners.

The proximal address may be held at a fixed length or increase in length as the cell grows. To distinguish these possibilities, we measured the length of the ectopic BASL domain at different developmental stages in a wild-type background. Domain length increased from ∼5 μm to ∼45 μm as cells increased in size, but at a rate lower than the rate of increase in cell perimeter (Figures 2E and 2F). This finding suggests that the proximal address does not have a fixed size but may be restricted through interactions with other factors in the cell, consistent with a model of polarity establishment involving intracellular partitioning [22].

The cytoskeleton has previously been associated with formation of cell polarity [23, 24]. To test if microtubules are required for positioning ectopic BASL, we destabilized microtubules with oryzalin before inducing BASL. In oryzalin-treated plants, ectopic BASL was still polarized (Figures S3A–S3G), suggesting that microtubules are not required for the polarization of BASL, similar to BRXL2 [2].

### Wild-Type Exhibits a Combination of Stomatal and Non-stomatal Polarity Fields

For a comparable stage, the proportion of BASL vectors outside the range of −80° to 80° was significantly higher for wild-type than for *spch* (Table S1). To determine whether the lower level of proximodistal coordination in wild-type was caused by more variable BASL polarity orientation in stomatal lineage cells, we imaged leaves expressing *BASL::GFP-BASL* [11]. BASL was asymmetrically localized within individual cells, as well as expressed in the nucleus (Figures 2O and 2P), as previously described [11, 16]. Although not obvious from inspection of a single leaf (Figure 2Q), when multiple leaves were pooled, proximodistal coordination was observed for BASL vectors in *BASL::GFP-BASL* (Figure 2R), as reported for BRXL2 [2]. BASL polarity was significantly less coordinated than for ectopic BASL in *spch* (Table S1). Wild-type background showed an intermediate distribution (Figure 2S and Table S1), suggesting that it reflects a mixture of two patterns: a strongly coordinated proximodistal pattern in non-stomatal lineage cells and a weaker coordinated pattern in stomatal lineage cells.

Two hypotheses may account for the weaker polarity coordination of the stomatal lineage. One is that the proximodistal address becomes reoriented in stomatal lineage cells, and ectopic BASL follows this pattern. Alternatively, stomatal lineage cells contain two addresses (i.e., two regions with BASL-interacting factors) that compete for ectopic BASL: a proximal address and an address specific to stomatal lineages.

### The Polarity Field Becomes Divergent during Development

**Figure 3:**
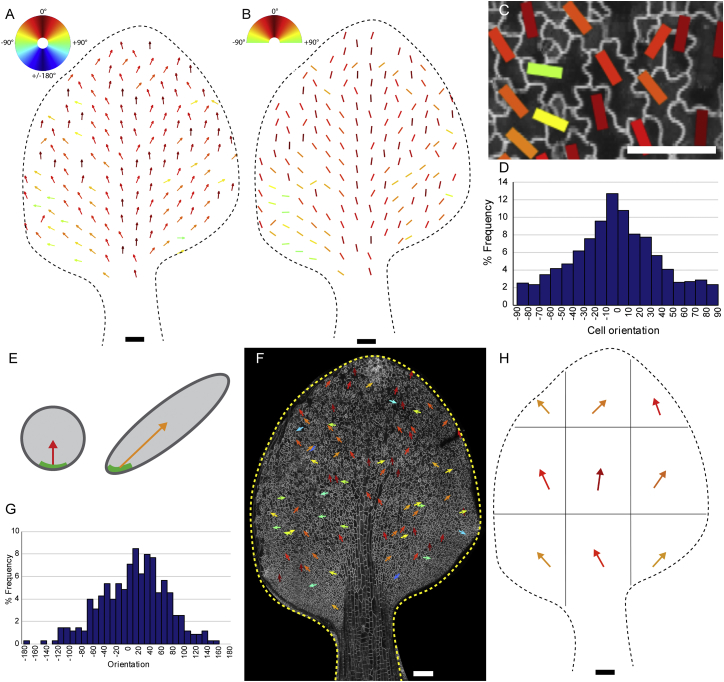
Ectopic BASL Polarity in a Wild-Type Background Becomes Divergent during Development and Is Present in Near-Isotropic Cells (A) Downsampled vectors in leaf of >800 μm width plotted according to color map. (B) Downsampled cell long axes for leaf in (A) plotted according to color map. Due to the long axes being tensors and not vectors, half of the color map is used. (C) Close-up of individual cell orientations for leaf in (B). Scale bar is 20 μm. (D) Orientation of cell long axis relative to leaf midline vector for leaf shown in (A) and (B). See also Figure S4. (E) Schematic of an isotropic cell with BASL localized to the proximal end (left) and an anisotropic cell where BASL polarity vector has become deflected, even though BASL position is unchanged (right). (F) Leaf in (A) with BASL vectors for cells with eccentricity <0.6 plotted. (G) BASL vector orientation for near-isotropic cells relative to leaf midline vector. Data pooled from 4 leaves of 800+ μm width. (H) Leaf in (A) divided into regions with average BASL vector orientations in each section shown and plotted according to color map in (A) See also Table S2. Scale bars are 100 μm except in (C).

To visualize the ectopic BASL polarity pattern more easily, larger leaves were downsampled by averaging vector orientations using a grid (Figure 3A). This analysis showed that vectors in the midvein region were highly coordinated in a proximodistal orientation, while those in the proximal lamina diverged away from the midvein toward the margin (Figure 3A). The ectopic BASL polarity field shows striking similarities with a polarity field previously proposed to account for orientations of growth [8]. In both cases, the polarity field becomes divergent at later stages of development. It has also been shown that the orientation of BRXL2 polarity is aligned with the orientation of subsequent growth [2]. These results suggest that polarity may provide orientation information to guide growth.

However, this interpretation is complicated because of the way polarity is assigned in relation to the centroid of the cell. For example, suppose BASL is proximal in a circular cell (Figure 3E, left). If that cell becomes elongated diagonally (either through growth or diagonal division), polarity will also become diagonal, even though there has been no change in the positioning of the BASL signal (Figure 3E, right).

To evaluate the effect of such cell shape anisotropy on polarity measurements, we determined the orientation of the long axis of each cell (Figures 3B and 3C). This showed that, on average, cells were preferentially elongated in a divergent pattern like that of the axial component of the ectopic BASL polarity field (Figures 3B and 3D), and this correlation was also confirmed by calculating the angle between the BASL vector and the cell long axis (Figure S4D). Thus, the divergent pattern of the ectopic BASL polarity field could be a consequence of cell shape anisotropy and the way polarity is assigned to cells.

To test this possibility, we analyzed the subset of cells from the wild-type background, which had a nearly isotropic shape (Figure S4). Ectopic BASL vectors of these near-isotropic cells showed a preferential proximodistal orientation, including the splayed-out pattern in the proximal region of the lamina (Figures 3F and 3G). The leaf was subdivided into regions, and average vectors from the isotropic cells were calculated. This also showed the splaying out across the lamina (Figure 3H and Table S2). Thus, the observed divergent proximodistal polarity field is not dependent on cell shape anisotropy, consistent with cell polarity orientation being a guiding factor rather than consequence of oriented growth.

### Ectopic BASL and PIN Mark a Common Polarity Field

The ectopic BASL polarity field resembles that for PIN1 localization at early stages of leaf development, except that whereas BASL localizes proximally, PIN1 in epidermal cells localizes distally [25]. It is possible that both polarity markers are part of a common system, with PIN involved in early establishment of polarity and ectopic BASL revealing a polarity that is maintained through to later stages. To determine the relationship between PIN1 and BASL localization, we developed a line with inducible *35S::mCherry-BASL* also expressing *PIN1::PIN1-GFP* so that both polarity markers could be visualized in the same cells, though BASL signal was less uniform across the tissue than in the inducible *35S::GFP-BASL* line.

**Figure 4:**
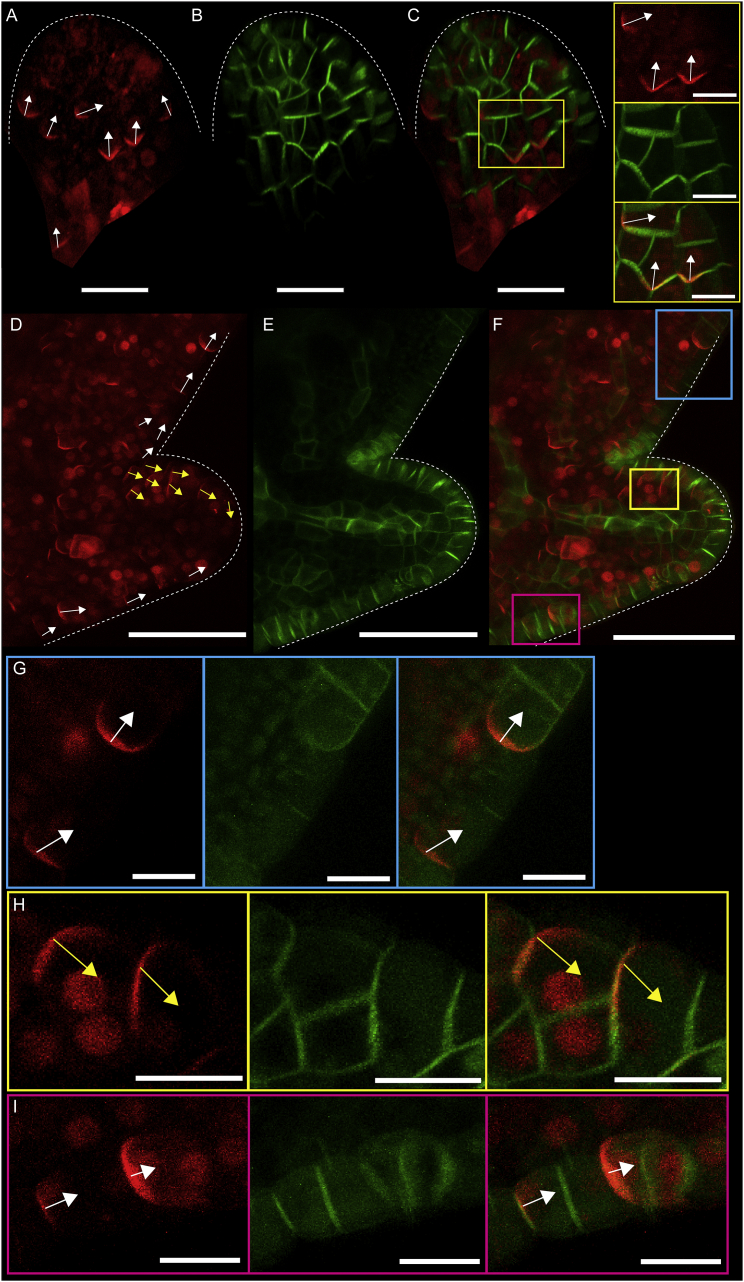
Ectopic mCherry-BASL Localizes to the Opposite End of Cells to PIN1 Mirroring Convergence and Divergence Points at Serrations (A) Induced *35S::mCherry-BASL* in leaf primordium. Arrows indicate manually assigned BASL polarity based on curvature of the BASL crescent. (B) *PIN1::PIN1-GFP* in same primordium as (A). (C) mCherry-BASL and PIN1-GFP signals combined. Yellow box indicates magnified region of leaf. Scale bars are 20 μm in (A)–(C) and 10 μm in close-up regions of (C). (D) Induced *35S::mCherry-BASL* at serration of leaf 5. Arrows are manually assigned, and yellow arrows highlight cells in which BASL is not proximally localized. (E) *PIN1::PIN1-GFP* in same serration as shown in (D). (F) mCherry-BASL and PIN1-GFP signals combined. Projections allow visualization of margin cells. Scale bars are 50 μm in (D)–(F). (G–I) Magnified regions of serration in (F) in blue (G), yellow (H), and magenta (I) boxes, respectively. z slices were selected to allow visualization of cells due to curvature of serrations. *35S::mCherry-BASL* (left), *PIN1::PIN1-GFP* (middle), and combined signals (right). White dotted lines indicate leaf outline. Scale bars are 10 μm in (G)–(I). See also Figure S3.

Induction of ectopic BASL in young leaf primordia showed that it localized to the proximal end of cells (Figure 4A) at a time when PIN1 was expressed. PIN1 had a broader distribution than ectopic BASL at this stage, making its polarity harder to assign (Figures 4B and 4C). Induction of ectopic BASL at later stages showed that co-expression with epidermal PIN1 expression was only observed in developing serrations (Figures 4D–4F). A region of reversed ectopic BASL polarity (yellow arrows) was seen at the distal edge of the serration, creating BASL convergence and divergence points (Figures 4D and 4F–4I). This BASL polarity pattern mirrors PIN1 convergence and divergence points previously described [5], with BASL localizing to the opposite end of the cell compared to that reported for PIN1. It has been shown that the PIN1 polarity pattern at serrations depends on a feedback loop involving auxin transport [5], suggesting that the polarity revealed by ectopic BASL is coupled to the same polarity-coordinating mechanism. To test the role of polar auxin transport in BASL localization, we grew seedlings on naphthylphthalamic acid (NPA), an auxin transport inhibitor, before inducing ectopic BASL. In NPA-treated seedlings, which exhibited root and leaf shape phenotypes [26, 27], ectopic BASL was still proximally localized (Figures S3H–S3M). The relationship between PIN, auxin, and ectopic BASL localization can vary, as ectopic BASL in roots has been shown to localize to the same end of cells as PIN or the opposite end, depending on the cell type and PIN family member [11].

### Origin of the Polarity Field

The coordination of the proximodistal polarity field throughout the leaf epidermis could be accounted for by mechanical and/or chemical mechanisms [22, 24, 28, 29, 30, 31]. The observation that mechanical stretching of a leaf can deflect the polarity field, as revealed by BRXL2 reorientation, indicates that tissue-wide mechanical forces can influence polarity [2]. However, the nature of polarity as a vector (with an arrow head) means that tissue stress, which has axiality but not polarity, is not sufficient to establish the directional aspect of the vector field [32, 33, 34]; thus, a stress gradient would be required [24]. Alternatively, a biochemical mechanism, such as flux sensing or cell-cell coupling, may underlie the coordination of the polarity field [22, 35, 36]. Such a mechanism has the advantage of being uncoupled from the stresses generated through differential growth [34].

In addition to influencing growth, the polarity field may also influence patterning and differentiation (e.g., trichomes [3, 4], stomatal patterning [16]). Orientation of both BASL and BRXL2 in stomatal patterning exhibits proximodistal coordination, albeit much weaker than observed for the non-stomatal lineage cells in the *spch* mutant. Although polarity is critical for stomatal spacing in *Arabidopsis* [16, 37], it is unclear why proximodistal coordination would be functionally important. It is possible that the coordination reflects evolutionary history rather than current function. Stomatal patterning mechanisms vary among plant species [11, 38, 39, 40, 41, 42]. By contrast, a proximodistal polarity field may be a highly conserved system for orienting tissue growth and transport [43, 44, 45]. Perhaps various elements of the proximodistal polarity system were co-opted for stomatal patterning in different plant lineages. For the lineage leading to *Arabidopsis*, co-option may have led to a polarity-switching mechanism and the evolution of BASL. This hypothesis would account for why BASL cross-reacts with the proximal address when ectopically expressed. Other plant lineages, such as grasses, which exhibit strong proximodistal coordination in stomatal patterning [38, 46], might represent different ways of co-opting elements of a fundamental proximodistal field.

Thus, the proximodistal field described here may have provided key elements that were co-opted during evolution for controlling patterns of differentiation and spacing. In addition, it may provide a conserved system for orienting growth in planar plant organs, similar to equivalent systems described for animal development [1].

## STAR★Methods

### Key Resources Table

**Table:** 

REAGENT or RESOURCE	SOURCE	IDENTIFIER
**Chemicals, Peptides, and Recombinant Proteins**		

Propidium Iodide	Sigma-Aldrich	Cat# P4170
Spe I	Sigma-Aldrich	11008943001
BspEI	New England Biolabs	Cat# R0540S
ccdB-resistant one-shot *E. coli*	ThermoFisher Scientific	Cat# A10460
Difco agar	Becton & Dickinson	Cat# 214030
Oryzalin	Sigma-Aldrich	Cat# 36182
N-1-naphthylphthalamic acid (NPA)	ChemService	N-12507

**Critical Commercial Assays**		

iDNA genetics copy number analysis	iDNA genetics	N/A

**Experimental Models: Organisms/Strains**		

Heat-shock inducible BASL (35S::GFP-BASL)	This paper	N/A
BASL::GFP-BASL	[11]	N/A
RFP-plasma membrane (pm-rb)	[47]	CD3-1008
RFP-plasma membrane with inducible 35S::GFP-BASL	This paper	N/A
*spch* mutant (*spch-1*)	[48]	N/A
*spch* with inducible BASL (35S::GFP-BASL)	This paper	N/A
HS::Cre	[17]	N/A
PIN1::PIN1-GFP	[49]	N/A
Heat-shock inducible 35S::mCherry-BASL with PIN1::PIN1-GFP	This paper	N/A
35S::TUA6-GFP	[50]	N/A

**Oligonucleotides**		

F_BOB_lox_speI (GGGACTAGTATCGCGGCCGCTTCGAAA)	This paper	N/A
R_BOB_lox_N (CTATACGAAGTTATACGCGTCTGT)	This paper	N/A
R3_BOB_lox_EcoRV (GGGATATCATAACTTCGTATAAAGTAT CCTATACGAAGTTATACGCGTCTG)	This paper	N/A

**Recombinant DNA**		

pBOB vector	[51]	N/A
TOPO4 vector	Invitrogen	N/A
pB7WGC2 vector	[52] (VIB Gent)	N/A
GFP-BASL entry clone	[11]	N/A
Destination vector with lox-HDEL:CyPET:NOS-Terminator-lox (Active blue destination vector)	This paper	N/A

**Software and Algorithms**		

cellfromleaves	This paper	Github; https://github.com/JIC-Image-Analysis/cells-from-leaves
cellsfromleavestagger	This paper	Github; https://github.com/JIC-Image-Analysis/cells-from-leaves-tagger
sampleArrows8	This paper	Github; https://github.com/JIC-Image-Analysis/cells-from-leaves/tree/master/matlab_scripts
cellLongAxisCorr7	This paper	Github; https://github.com/JIC-Image-Analysis/cells-from-leaves/tree/master/matlab_scripts
VolViewer		http://cmpdartsvr3.cmp.uea.ac.uk/wiki/BanghamLab/index.php/Software#Viewing_and_measuring_volume_images:_VolViewer

### Contact for Reagent and Resource Sharing

Further information and requests for resources and reagents should be directed to and will be fulfilled by the Lead Contact, Enrico Coen (enrico.coen@jic.ac.uk).

### Experimental Model and Subject Details

#### Growth conditions

*Arabidopsis* plants were grown on plates containing MS media (0.441% Murashidge & skoog including vitamins, 1% (w/v) glucose, 0.05% (w/v) MES, 1% Difco agar, pH to 5.7) and relevant antibiotic selection. Seeds were gas or surface sterilized and stratified in the dark at 4°C for 3 days, then grown at 20°C in long day conditions (16 hours light, 8 hours dark). Leaves were taken from plants up to 9 days after stratification for imaging and analysis.

#### Genetic material

The transgenic lines *spch-1* [48], *HS::Cre* [17], *BASL::BASL-GFP* [11], RFP-PM [47], *PIN1::PIN1-GFP* [49] and *35S::TUA6-GFP* [50] are in the Col-0 background.

### Method Details

#### Construction of transgenic plants

We used Gateway cloning to construct heat-shock inducible *35S::GFP-BASL* line which required a destination vector and an entry vector. We made a destination vector (which we refer to Active Blue destination vector) containing a 35S promoter in front of a CyPET:HDEL fluorescent marker and a Nos terminator flanked by lox sites. These lox sites will later allow heat-shock recombination to remove the fluorescent marker so that the 35S promoter drives a downstream gene of interest.

The Active Blue destination vector was made using a pre-existing Gateway vector, pB7WGC [52] and the pBOB [51] vector. The procedure involved 2 steps. In the first step, a 1175 bp fragment containing lox-HDEL:CyPET:NOS-Terminator-lox was cloned from pBOB and flanked with SpeI and EcoRV sites using a 2-step PCR, involving the primers F_BOB_lox_speI and R_BOB_lox_N, then primers F_BOB_lox_speI and R3_BOB_lox_EcoRV. The PCR product was then cloned into TOPO4. In the second step, the pB7WGC2 vector was digested with SpeI and BspEI, to excise a 1175 bp fragment containing ECFP, and replaced with the fragment cloned from pBOB vector (cut out from the TOPO4 vector using SpeI and BspEI). The ligation product was transformed into ccdB-resistant one-shot E.coli.

To introduce GFP-BASL into the destination vector, an LR reaction (Invitrogen) was carried out using the Active Blue destination vector and an entry clone containing GFP-BASL [11].

For transformation of *Arabidopsis* plants, *Agrobacterium tumefaciens* strain GV3101 and floral dip method were used [53] to dip into HS::Cre [17] containing plants. Three independent lines were obtained showing the same pattern. The line used is a single copy, single insert line (iDNA genetics).

The inducible *35S::BASL-GFP* line was crossed to the heterozygous *spch-1* mutant plants and offspring containing *spch-1* and inducible *35S::BASL-GFP* were selected by phenotype and growing on selective plates (Basta for *35S::BASL-GFP*, Kanamycin for *HS:Cre*). The inducible *35S::BASL-GFP* line was crossed to the RFP-PM line [47] and offspring containing RFP-PM and inducible *35S::BASL-GFP* were selected by growing on selective plates and screening for RFP.

To make the line with inducible *35S::mCherry-BASL* and *PIN1::PIN1-GFP*, we generated a construct containing inducible *35S::mCherry-BASL* and *HS::Cre* using golden gate cloning and dipped [53] this into *PIN1::PIN1-GFP* [49] containing plants. The line used contains 2 copies (iDNA genetics). The 35S::loxmCherry-BASLloxCyPET-HSP18::CRE-35S::Basta-35S::CyPET-RC12A (called inducible *35S::mCherry-BASL* for simplicity) construct was created by Golden Gate cloning in the vector pAGM4723 (Addgene #48015) as described by Weber et al. (2011). Level 0 modules were domesticated to remove BsaI, BpiI and DraIII restriction sites and synthesized synthetically. To generate the lox-flanked mCherry Level 1 module we adapted the standard Golden Gate protocol to incorporate an additional assembly step, termed Level 0.5. Here the vector backbone EC10161 is opened by the enzyme Esp3I to allow the insertion of Level 0 modules cut by BsaI, just as for standard Level 1 cloning. This generates loxP flanked modules in the ‘U’ position suitable for use in subsequent Level 1 assembly. Sequences to be used in loxP-flanked modules were domesticated to be free of Esp3I sites in addition to BsaI, BpiI and DraIII recognition sites. Plasmid maps are available on request.

#### Propidium iodide staining

To stain leaves with propidium iodide, leaves were submerged in a 2.5 μg/ml propidium iodide solution (PI - Sigma) for at least 15 minutes before imaging.

#### Confocal microscopy

For confocal imaging, leaves (typically first true leaf other than for serrations) were placed in water under a coverslip, or in the optical imaging chamber [54]. Imaging was performed using a x10 or x20 dry lens, or x40 oil lens, on a Leica SP5 confocal microscope equipped with Leica HyD Hybrid detectors, or a Zeiss 780. For imaging GFP, argon ion (488 nm) excitation laser was used, collected at 495-530 nm. For PI, mCherry and RFP, 561 nm excitation was used, collected at 625-690 nm for PI, 575-630 for RFP and 600-620 nm for mCherry. Leaves were staged according to leaf width and were typically imaged 48-hours after heat-shock. Seedlings were typically heat-shocked for 20 minutes to induce BASL across the entire lamina, and 3 mins to induce sectors.

To image *35S::GFP-BASL* appearing after induction, 7 day old seedlings were heat-shocked for 20 mins and placed in an imaging chamber with media as described in [55]. Leaves were imaged every hour using a Zeiss 780 confocal microscope, with the settings described above.

#### Oryzalin treatment

Oryzalin was added to 6-day old seedlings (*35S::GFP-BASL* line described above and *35S::GFP-TUA6* as control line) at a concentration of 20 μM. Seedlings expressing *35S::TUA6-GFP* have previously been described [50]. *35S::TUA6-GFP* seedlings confirmed microtubules had depolymerized after 4 hours and seedlings were heat-shocked to induce BASL expression. Plants were imaged 48 hours after heat-shock, with *35S::GFP-TUA6* confirming the absence of microtubules.

#### NPA treatment

*35S::GFP-BASL* seedlings were grown on media containing 100 μM NPA, or an equivalent concentration of DMSO. Seedlings were heat-shocked 2DAS and leaves imaged 3 days later. Propidium iodide staining (described above) was used to visualize cell outlines.

### Quantification and Statistical Analysis

#### Cells-from-leaves and cells-from-leaves-tagger software

For assigning BASL vectors, Python software was developed using jicbioimage [56]. It used the cell outline channel (either plasma-membrane marker or PI stain) from the confocal stack to make a projection of the leaf surface. The leaf surface projection was used to reduce noise by only extracting signal from the volume occupied by the leaf. The cell outline channel extracted from the leaf surface was then used as input to the watershed algorithm. Leaf-specific parameters allowed the surface and segmentation to be customised according to intensity and quality of image. The centroid for each cell was calculated. BASL signal was also extracted from the cell surface.

To avoid bias arising from knowledge of the orientation and position of a cell within the context of the whole leaf, each segmented cell was presented to the user in isolation, randomly rotated in one of four orientations (0, 90, 180, 270 degrees). For each cell the user then selected a point in the middle of any visible BASL crescent, or chose to skip a cell if there was a complication (i.e., if the signal was not easy to identify, or the cell segmentation was incorrect). For a sample leaf image, out of 162 cell assignments of BASL, 157 were based on three-way junctions, and 5 were based on concavity of the BASL signal. The tool produced a directory of JSON files and corresponding image files, recording the BASL orientation in separate files for each cell, along with an image of the cell segmentation. Lastly, BASL vectors were transformed back into the coordinate system of the whole leaf, and written out to a CSV file along with the coordinates of each cell centroid.

#### sampleArrows8 software and cellLongAxisCorr7 software

We developed two MATLAB scripts, one to allow us to quantify the BASL vector field (cellLongAxisCorr7.m) and one to visualize it in a more informative way (sampleArrows8.m).

One script developed, sampleArrows8, is for visualizing BASL vectors on the leaf, and down-sampling them. This script uses a leaf image and .csv file of BASL vectors (produced by ‘cells from leaves’). The user identifies the leaf midline which is used to rotate the leaf image and BASL vectors to allow the image to be vertically oriented. The script contains various processing and display options, but it is frequently used to display the original BASL arrows on the leaf, colored by orientation with respect to the leaf midvein. The color of each arrow is determined by a color map, where 0 degrees represents the proximodistal orientation.

There can be a lot of BASL vectors on a leaf, with some areas having a very high density of points. BASL vectors can therefore be down-sampled to reduce the total number of vectors displayed and to give a more even spread of BASL vectors across the leaf. Down-sampling uses a triangular grid of points placed over the leaf. For each vertex of the grid, vectors within the distance Maxdist are averaged. A parameter, neighborThreshold, ensures that down-sampled BASL vectors are only displayed for samples that exceed the threshold number of BASL vectors.

This script can also be applied to cell orientations. This is achieved by gathering cell orientations within a certain radius, normalizing and superimposing them onto the same axis, and then performing principle component analysis (PCA) on that cloud of points.

We also developed a script named cellLongAxisCorr7, which quantifies the BASL vector field. This script calculates various angles: orientation of cell axis, angle between BASL vector and its cell axis, and angle between BASL vector and leaf midvein axis. This script uses an image of the leaf and the directory of JSON files to rotate the cells back to their original orientation and cell masks are derived, allowing cell eccentricity (ratio of the distance between the foci of the ellipse fitted to a cell and its major axis length), centroid and orientation of the long axis of the cells to be determined.

For each cell, three angle measurements are made: the angle between the BASL vector (from the JSON files) and the cell long axis, angle between the BASL vector and the leaf midline axis (specified by the user), and the angle between the cell long axis and the leaf midline axis. Subsets of data can be selected by specifying lower and upper threshold values in the script parameters (for cell eccentricity and orientation relative to the leaf). The script displays the orientation information as histograms and also writes it out to CSV files for further analysis. To select the near-isotropic cells, we first calculated eccentricity of the segmented cells (cell eccentricity is the ratio of the distance between the foci of the ellipse fitted to a cell and its major axis length). Cells with an eccentricity of less than 0.6 were considered near-isotropic.

Further documentation is found in both sampleArrows8.m and cellLongAxisCorr7.m. These scripts also contain a detailed explanation of each of the input parameters.

#### Additional image analysis

BASL crescent length and cell perimeter were calculated by clicking round the BASL signal and cell outline using Fiji [57] measure tool for cells of different sizes. To determine average BASL vector orientations for near isotropic cells in regions of the leaf, cellLongAxisCorr7 was used with a maximum eccentricity value of 0.6, and vectors were visualized on the leaf using sampleArrows8. The leaf was then subdivided into 9 regions and vectors measured in each region measured using Fiji angle tool.

Statistical comparison of BASL vector distributions between genotypes, was performed using chi-square tests (df = 1, p values less than 0.01 were considered significant), comparing frequency of BASL vectors within or outside the range of −80° to 80°, in pairwise tests.

For z stacks of leaves expressing *PIN1::PIN-GFP* and *35S::mCherry-BASL*, images were rendered in 3D using Volviewer. For serrations, Fiji was used to create maximum projections for visualization. Specific ranges of z-slices were used to allow visualization of specific cells.

### Data and Software Availability

The custom code that implements the segmentation and random orientation pipeline (cells-from-leaves) is available at: https://github.com/JIC-Image-Analysis/cells-from-leaves.

The tool for visualizing cell segmentations and selection of BASL signal in the cell (cells-from-leaves-tagger) is available at: https://github.com/JIC-Image-Analysis/cells-from-leaves-tagger.

MATLAB software for visualization of vectors and angle calculation available at https://github.com/JIC-Image-Analysis/cells-from-leaves/tree/master/matlab_scripts.

VolViewer available for download from http://cmpdartsvr3.cmp.uea.ac.uk/wiki/BanghamLab/index.php/Software#Viewing_and_measuring_volume_images:_VolViewer.

### Additional Resources

Plasmid maps for lines generated and raw data for leaf images available on request.

## Supplemental Information

Document S1. Figures S1–S4 and Tables S1 and S2

Document S2. Article plus Supplemental Information
