# Supplementary material for: Ectopic BASL Reveals Tissue Cell Polarity throughout Leaf Development in Arabidopsis thaliana
Source: Curr Biol. 2018 Aug 20;28(16):2638–2646.e4. doi: 10.1016/j.cub.2018.06.019 (PMC6109230; doi:10.1016/j.cub.2018.06.019)
Supplement: Document S1. Figures S1–S4 and Tables S1 and S2 [file mmc1.pdf]

Current Biology, Volume *28*

Supplemental Information

Ectopic BASL Reveals Tissue Cell Polarity throughout Leaf Development in *Arabidopsis thaliana*

Catherine Mansfield, Jacob L. Newman, Tjelvar S.G. Olsson, Matthew Hartley, Jordi Chan, and Enrico Coen

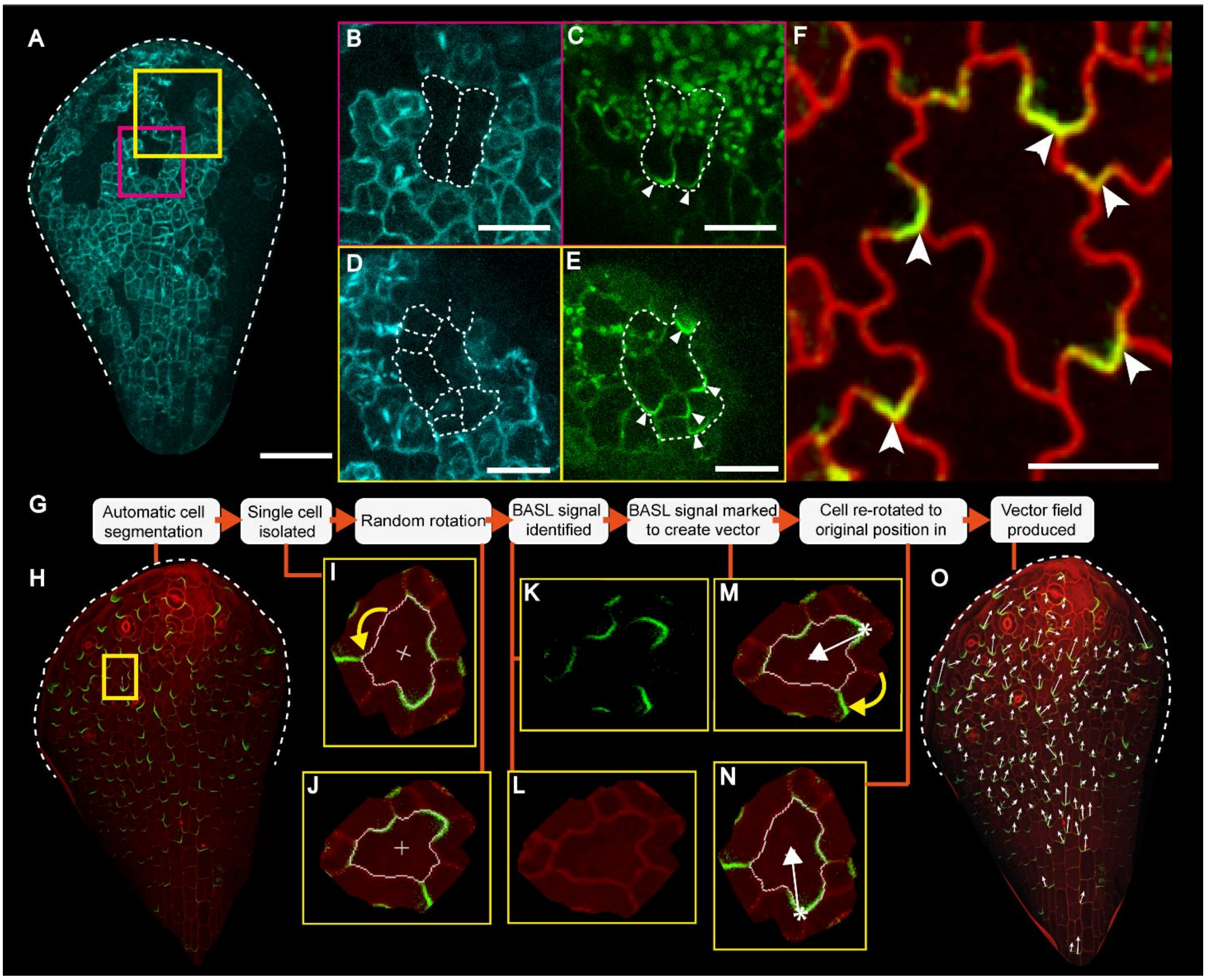

Figure S1. Vectors can be added to each cell using semi-automated software to show position of BASL signal with respect to the cell centroid. Related to Figure 1 and Figure 2.

(A) 35S::GFP-BASL induced in small sectors across the leaf (magenta and yellow boxes) composed of a few cells. (B and D) Absence of ER-tagged CFP indicates where sectors are induced. (C and E) GFP-BASL signal in the sectors localised to the proximal end of the cells (BASL signal indicated by white arrowhead). White dashed line indicates leaf outline in A, cell outlines in sectors in B and D and sector outlines in C and E. (F) 3-way junctions between cells (white arrow head) show which cell the 35S::BASL-GFP signal belongs to. Scale bars are 100 µm in A and 20 µm in B-F. (G) Image processing pipeline (using 'cellfromleaves' and 'cellsfromleavestagger' software). (H) Raw confocal data is automatically segmented. (I) Individual cells are identified and position of centroid (cross) is extracted. Yellow arrows indicate cell rotation. (J) Individual cell is randomly rotated in one of 4 orientations. (K and L) BASL signal is identified from merged image of cell and separate colour channels for clearer visualisation. (M) BASL signal marked by hand (indicated by asterisk) to create vector, indicated by white arrow. (N) Cell and vector are rotated back into original position. (O) Process repeated for every segmented cell to produce a vector field for the leaf. White dashed line in H and O indicates leaf outline.

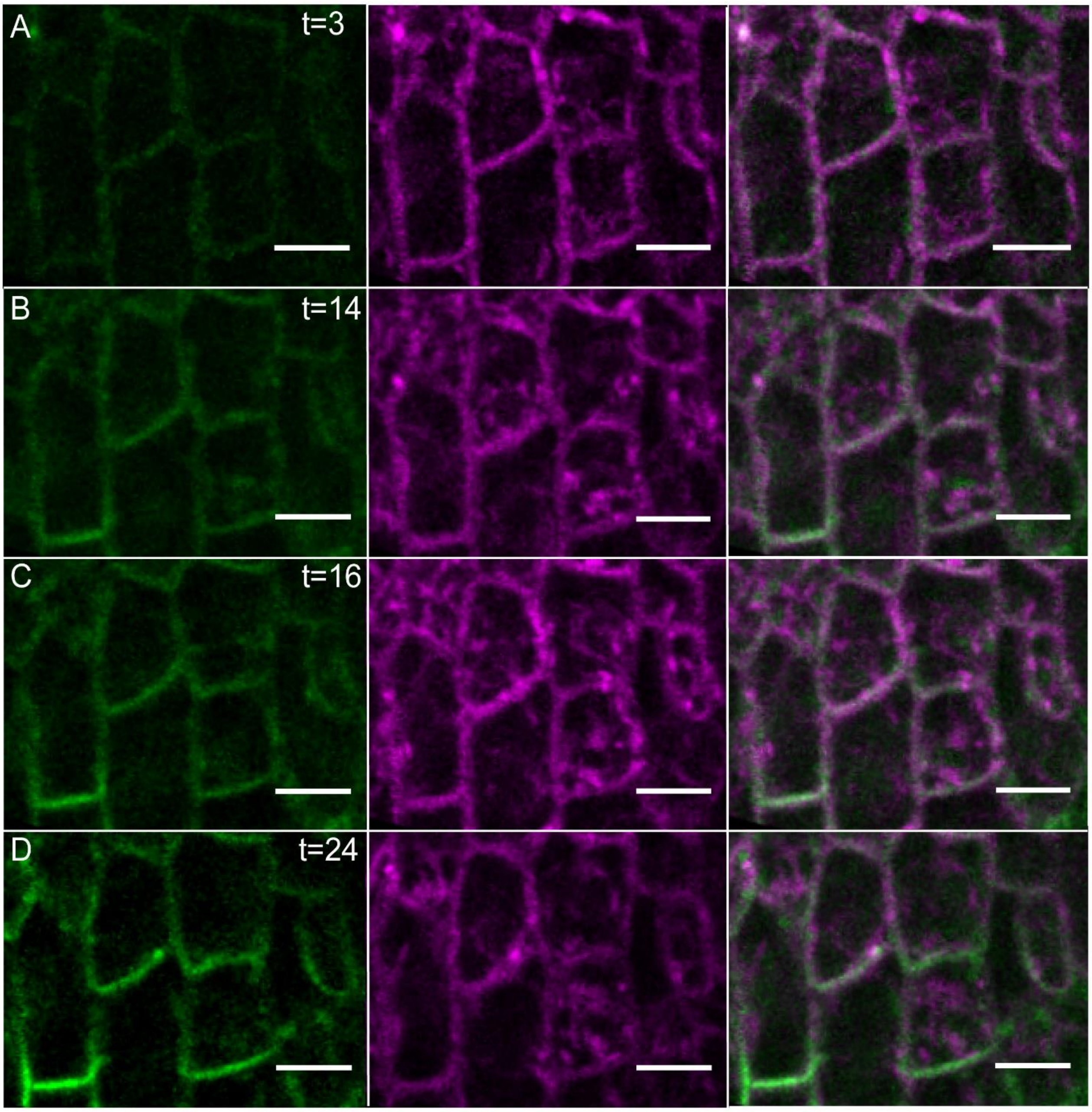

Figure S2. Time lapse imaging after 35S::GFP-BASL induction. Related to Figure 2.
(A) 35S::GFP-BASL induction in a wild type background following heat-shock at 3 (A), 14 (B), 16 (C), and 24 (D) hours after heat-shock. At 3 hours, no BASL is seen, comparable to uninduced leaves. Left hand panels show GFP-BASL expression appearing at the proximal end of cells with increasing intensity. Middle panels show ER-localised CFP outside the lox sites, coloured magenta for clear visualisation. Right hand panels show combined GFP-BASL and ER-CFP channels. Scale bars are 10 µm. Images are maximum projections of multiple z-slices to accommodate movement of the leaf during imaging.

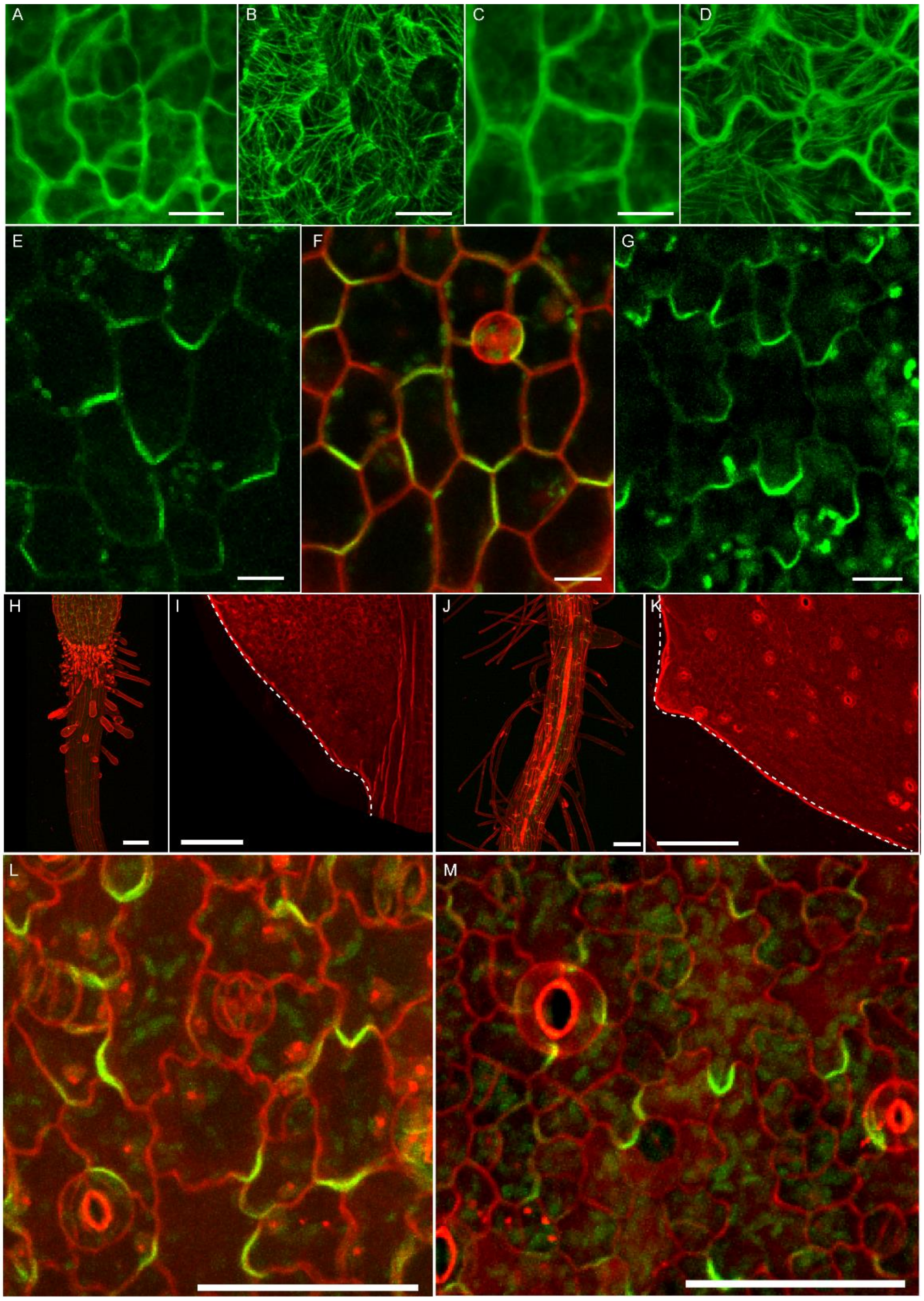

Figure S3. 35S::GFP-BASL localisation remains proximal in cells treated with oryzalin and NPA. Related to Figure 4.
(A) 35S::Tubulin-GFP after treatment with 20 µM oryzalin for 4 hours showing microtubules depolymerised. (B) 35S::Tubulin-GFP after equivalent treatment to A with DMSO. (C) 35S::Tubulin-GFP after treatment with 20 µM oryzalin for 2 days showing microtubules still depolymerised. (D) 35S::Tubulin-GFP after equivalent treatment to C with DMSO. (E, F) Examples of 35S::GFP-BASL induced in leaves treated with 20 µM oryzalin for 2 days. BASL signal is proximally localised. Cell wall stained with PI (red) in F. (G) 35S::GFP-BASL proximally localised in leaves treated with DMSO equivalent to E,F. Scale bars 10 µm in A-G. (H) Root of NPA (100µM) treated seedlings did not produce lateral roots or fully developed root hairs. (I) Leaf outline of NPA (100µM) treated seedlings did not produce a wild-type serration. (J) Root and (K) leaf outline of DMSO treated seedlings showing lateral roots and root hairs, and serration respectively. Dotted white line indicates leaf outline. Scale bars 100 µm in H-K. (L) 35S::GFP-BASL induced in leaves grown on 100 µM NPA remained proximal. (M) 35S::GFP-BASL induced in leaves grown on DMSO control. PI staining shows outlines. Scale bars in L-M are 50 µm.

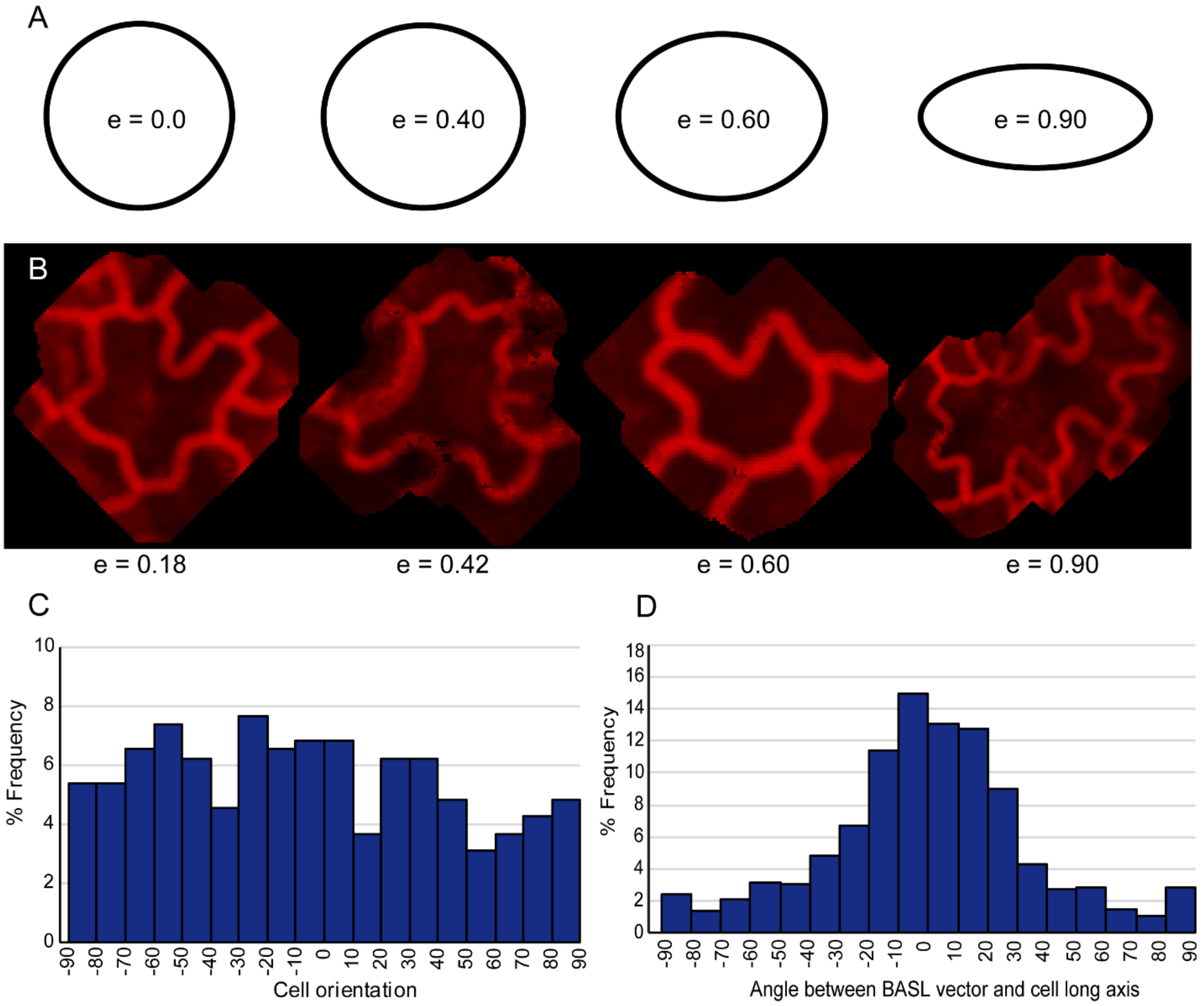

Figure S4. A cell with eccentricity of less than 0.6 was considered near isotropic. Related to Figure 3.
(A) Schematic showing ellipse eccentricities. An eccentricity of less than 0.6 was considered to be near isotropic. (B) Examples of cell eccentricities showing cells regarded as near isotropic (except cell on far right with an eccentricity of 0.9). Cell outlines shown in red by RFP-PM. (C) Histogram showing cell long axis orientation relative to the midline vector for near isotopic cells (total from 4 leaves greater than 800 µm in width), showing that this subset of cells has no preferential orientation. (D) Histogram showing frequencies of angle between BASL vector and cell long axis (for leaf shown in Figure 3A), indicating correlation between BASL vector and cell long axis orientation.

**Genotype comparison**	**n = BASL vectors inside -80 to 80 range**	**n = BASL vectors outside -80 to 80 range**	**n = leaf number**	**Chi-squared (df=1)**	**P value**
WT vs *spch*	WT = 6025<br>*spch* = 3169	WT = 1013<br>*spch* = 278	WT = 33<br>*spch* = 26	85	$p < 10^{-5}$
WT vs Native	WT = 6025<br>Native = 888	WT = 1013<br>Native = 431	WT = 33<br>Native = 22	260	$p < 10^{-5}$
spch vs Native	*spch* = 3169<br>Native = 888	*spch* = 278<br>Native = 431	*spch* = 26<br>Native = 22	456	$p < 10^{-5}$

Table S1. Two-way chi-squared tests for comparing BASL vector orientation across genotypes. Related to Figure 2. Chi-squared tests comparing BASL vector orientation across genotypes, based on number of vectors within the range -80° to +80° compared to outside this range. 'WT' refers to inducible 35S::GFP-BASL in a wild-type background, '*spch'* refers to inducible 35S::GFP-BASL in a speechless background, and 'native' refers to BASL::GFP-BASL. Note that as the distributions were not normal we used a non-parametric test.

Top left	Top middle	Top right	Mid left	Mid middle	Mid right	Bottom left	Bottom middle	Bottom right
-41.1	38.9	-19.3	-24.1	4.5	35.3	-40.5	-28.0	42.8
-45.3	8.4	19.6	-57.4	-3.5	64.3	-60.4	-4.2	66.6
3.7	11.6	-15.9	-28.0	-20.6	7.2	-36.6	-15.5	19.6
-1.1	3.5	-26.6	-29.3	-13.2	25.0	-83.0	10.5	86.9

Table S2. Average vectors from isotropic cells in regions of additional subdivided leaves shown. Related to Figure 3. The first row of data shows average vectors for regions of leaf shown in Figure 3H, the bottom 3 rows show average vectors for leaves in 3 additional leaves with widths greater than 800 µm.
